# Impacts of Food Insecurity on Child Development: Strengthening the Role of Childcare

**DOI:** 10.3390/nu17152427

**Published:** 2025-07-25

**Authors:** Emma G. Casey, Adam Winsler

**Affiliations:** Department of Psychology, College of Humanities and Social Sciences, George Mason University, Fairfax, VA 22030, USA; egregor5@gmu.edu

**Keywords:** child welfare, child day care centers, early childhood nutrition, early childhood education, hunger, food assistance, nutrition policy, preschool, psychosocial development

## Abstract

In 2023, the USDA reported that 17.9% of U.S. households with children were food insecure, meaning they had limited or uncertain access to adequate food. However, there is evidence that far more children experience food insecurity than is currently being reported, and the effects of that insecurity on child health and development are broad and far-reaching. Childcare and early childhood education centers are particularly well-positioned to make a difference yet are often not discussed in the scientific literature. Childcare arrangements provide meals and snacks to the children they serve, buffer the effects of food insecurity by supporting children’s cognitive and social–emotional development, and provide an important point of intervention for food-insecure families. In this report, we unpack the definition of food insecurity and who is considered food insecure, review how food insecurity impacts child health and development across physical, social–emotional, and cognitive domains, and explore the evidence behind childcare’s role in addressing childhood food insecurity. Additionally, we make recommendations for policy and practice, advocating for a multi-stakeholder approach, with a special focus on how childcare providers can change to best combat children’s food insecurity.

## 1. Introduction

Food insecurity in the U.S. is rising and is particularly high among households with children [1]. Mounting evidence demonstrates that food insecurity has negative impacts on young children’s health and development, above and beyond that of poverty and other risk factors [2,3]. Action is urgently needed to reduce the rates of food insecurity in the US. Authoritative literature reviews and a meta-analysis on food insecurity in early childhood as well as a review of interventions to combat food insecurity highlight the importance of food assistance programs like the National School Lunch Program, the Supplemental Nutrition Assistance Program (SNAP) and the Special Supplemental Nutrition Program for Women, Infants, and Children (WIC), social welfare payments (e.g., unemployment benefits and housing assistance), and strategies to address food deserts [3,4,5]. While the importance of these programs cannot be understated, the role of childcare in addressing food insecurity and mediating its impact on health and development is not often discussed.

Many childcare arrangements, including family home-based childcare, childcare centers, and preschool programs, are well-positioned to address child food insecurity and its effect on child development. Childcare is uniquely situated in families’ ecosystems as a third space that families visit daily. Additionally, not only do childcare providers supply cognitive and social–emotional enrichment, possibly mediating the effects of food insecurity on child development, but they are also responsible for feeding children, sometimes with food that they provide. Indeed, research suggests children consume more nutrient-dense foods at childcare than they do at dinner time at home [6], and that some childcare arrangements can reduce household food insecurity [7]. However, to date, there has not been a robust, scientific discussion of the ways in which childcare arrangements can be leveraged to combat food insecurity in U.S. households with children.

Our primary aim is to explore how childcare may be a point of intervention on food insecurity, highlight ways those interventions could be made more effective, and provide recommendations for multi-stakeholder action. In Section 2, we provide important background on the definition and measurement of food insecurity and then summarize its effects on child health and development in Section 3. In Section 4, we review the evidence on the effectiveness of childcare arrangements in mitigating food insecurity and its effects on health and development and discuss the critical policy and practice barriers that prevent childcare centers from being more effective. We conclude with implications for researchers, advocates and policymakers, and childcare providers in Section 5. We hope that this review can serve as an evidence-based road map to realizing the full potential of childcare arrangements in supporting food security and child health and development in the US.

## 2. Definition and Measurement of Food Insecurity in the US

According to the U.S. Department of Agriculture (USDA), food insecurity is defined as “limited or uncertain availability of nutritionally adequate and safe foods, or limited or uncertain ability to acquire acceptable foods in socially acceptable ways” [8]. The USDA has measured the national prevalence of food security with the Household Food Security Survey Module (HFSSM), a series of 10 questions (18 for families with children) that is part of the Current Population Survey that has been distributed annually for over two decades [8,9]. The HFSSM has also been widely utilized or adapted by researchers in the US [3] and globally [4]. Questions ask about the frequency (often/sometimes/never) of food running out before there is money to obtain more, changes to eating behavior and quality of food purchased as a result of not having enough money for food (e.g., skipping meals), worry about having enough food, and the effects of not eating enough (e.g., hunger and weight loss). Depending on the number and types of items respondents report experiencing, families are sorted into four categories of food security: very low, low, marginal, and high food security. Very low food security reflects experiences of insufficient food quantity and quality; low food security reflects experiences with reduced food quality but not quantity; marginal food security reflects occasional difficulty or anxiety about accessing food, but not insufficient food quality or quantity; and high food security reflects no difficulty or anxiety about accessing food [1]. Very low and low food security are considered “food insecure”, while marginal food security and food security are considered “food secure” [8].

Using the HFSSM in the annual Current Population Surveys, the USDA reports that in 2023, 17.9% of households with children in the US were food insecure, a marked increase from 12.5% in 2021. Food insecurity is also disproportionately experienced by people of color and those with near-poverty incomes; 22.4% of Black, non-Hispanic households, 20.8% of Hispanic households, and 32% of low-income households experience food insecurity [1]. Further, it is likely that the true prevalence of food insecurity is one to three percentage points higher due to respondents under-reporting their food security status [10], and may be even higher if the experience of food insecurity is conceptualized more broadly [11,12].

Indeed, although the HFSSM has been widely implemented, creating a cohesive and easy-to-synthesize body of literature, the USDA’s definition and measurement of food security and insecurity are limited. The HFSSM primarily concentrates on financial aspects of food insecurity. While there is no globally agreed-upon grounding theory of food security [4], most scholars recognize that inadequate money for food is just one domain of food insecurity; other domains include inadequate quality, cultural appropriateness, nutritional adequacy, accessibility, and the inability to access food via socially acceptable avenues [4,11,13]. The HFSSM does not ask specifically about cultural appropriateness, personal acceptability, or nutritional quality. It does not assess the degree to which food is available (i.e., if households live in a food desert), if food is obtained from assistance programs, food banks, or other arrangements (e.g., relying on a relative, neighbor, or a food pantry), or if it is obtained through methods considered unorthodox or socially unacceptable (e.g., dumpster diving). In fact, the USDA did not detect an increase in food insecurity after the onset of the COVID-19 pandemic; its estimates of food insecurity remained unchanged from 2019 to 2020 even though food pantry use is known to have increased considerably during that time [14].

In its assessment of food security status, the HFSSM also does not adequately measure or prioritize information related to uncertainty or worry about food, which is an important factor in understanding how food insecurity may impact child development outcomes. There is evidence of a positive, bi-directional relationship between food insecurity and depression [15,16]. Mounting evidence suggests that parental mental well-being may be a mediator between food insecurity and child development outcomes [17]. For example, parental stress from food insecurity indirectly impacts children’s self-regulation in preschool [18].

Even without concurrent food insufficiency, worry about food is still a part of the food insecurity spectrum [13] and has real consequences for children and their families [19]. The USDA considers these households to be marginally food secure, and includes them in their rates of food security. However, marginally food-secure households are more similar to food-insecure families than food-secure households [11]. Because families transition in and out of food security categories frequently [20,21], marginally food-secure families are at risk for a reduction in food intake or quality in the near future. Once anxiety about food is experienced, eating patterns and dietary quality are affected even when food is available [22,23]. For example, one woman interviewed said, “I can’t just walk to the refrigerator and just get what I want, no. I mean, there’s stuff out there to eat, but you think of tomorrow” [22]. Marginal food security may also have implications for child development; one study found that endorsing just one or two items on the HSSFM was as important as endorsing many items when it came to predicting impacts on math learning in kindergarten [24], and another found that persistent marginal food security negatively impacted children’s self-control and interpersonal skills [20]. These studies call into question the USDA’s practice of categorizing marginally food-secure households as food-secure households in their calculations and reports.

## 3. Impact of Food Insecurity on Early Child Development

Recent systematic reviews and meta-analyses have found that food insecurity has profound, broad effects on health and development across life stages [2,3]. The purpose of this paper is not to replicate recent systematic reviews and meta-analyses published on the association between food security and child development, but rather to provide a brief overview of food insecurity’s impact on child health and development in high-income countries, including physical health, dietary quality, and child development, including motor development, social–emotional development, and cognitive development, with a focus on the preschool years. This section also highlights important nuances, including when in childhood food insecurity is experienced, and specific mechanisms that may explain the association. Food security is strongly associated with poverty, which can have similar effects on children’s health and development [25]. This section describes the effect of food security above and beyond (and controlling for) the effects of family income and poverty in general.

### 3.1. Physical Health and Dietary Quality

Food-insecure children between 2 and 17 years old have worse overall health, including a higher prevalence of asthma, depressive symptoms, and visits to the emergency room compared to food-secure children [26]. The evidence linking food insecurity to cardiometabolic outcomes and risk factors (e.g., obesity, diabetes, heart disease) is robust in adults but mixed in children [19,27,28]. Some studies suggest that food insecurity in early childhood is associated with obesity both concurrently [29] and several years later in life [30,31], while others suggest no relationship [19] or an association with being underweight years later [32]. Lam et al. (2025) found that food insecurity in the preschool years is associated with poorer cardiovascular health in young adulthood, especially among families who did not participate in SNAP [31]. Interestingly, Zhu et al. [32] found that while food insecurity in kindergarten was associated with being underweight in eighth grade, food-insecure children in third, fifth, and eighth grade were more likely to be overweight compared to their food-secure peers. These findings suggest that the timing of food insecurity may have differential effects on weight, and that preschool may be a sensitive period for the effect of food insecurity on weight trajectories.

Perhaps more important than how food insecurity impacts weight is how it affects dietary quality and nutrient adequacy, as these factors may impact health across all domains. Food-insecure children are less likely to eat enough of some recommended food groups such as fruits and whole grains [33,34], and more likely to eat added sugars [35]. Additionally, food insecurity is associated with lower micronutrient adequacy [23] and iron deficiency anemia in U.S. children [36]. Nutritional adequacy—energy, protein, fatty acids, and micronutrients such as iron and iodine—is critical for neural development and central nervous system processes, particularly in infants 6 months to 3 years old, a time of rapid brain development [37]. Deficiencies at this age may have long-lasting effects; iron deficiency anemia in infancy may result in impaired motor, social–emotional, and cognitive development in the preschool years and beyond [38].

Inconsistent food availability may create problematic eating and feeding patterns. Food insecurity has been characterized by cycles of food restriction when food is not available and compensatory overeating when it is. Food-insecure families may experience a temporary increase in food availability when they receive their monthly SNAP benefits, but then deplete it before the end of the month [39]. Parents cite the end of the month and school summer holidays as particularly difficult times for feeding their family [39]. One study found that by the second week of receiving their monthly distribution, families only had one quarter of their benefits remaining [40]. Such experiences in childhood may contribute to the development of unhealthy eating patterns and attitudes that are maintained much later in life [41].

Food-insecure parents are also less likely to use responsive feeding practices [42,43,44]. Responsive feeding practices (such as responding to child’s hunger and satiety cues, encouraging eating in non-coercive ways, and modeling healthy eating) are linked to healthier weight and dietary outcomes, whereas controlling feeding practices (such as pressuring children to eat, using food as a reward, or overly restricting food) are inversely associated with such outcomes [45,46,47]. Parents, fearing that their child is not receiving enough food, may try to persuade their children to eat either with direct commands or via food rewards (e.g., promising a sweet treat if they eat their dinner) [39,48]. Poor food resource management skills—skills that help parents make the most out of their food supply, like planning meals prior to grocery shopping—may also result in parents using more controlling feeding practices [49].

### 3.2. Developmental Delays and Motor Development

Studies consistently demonstrate that food insecurity is associated with developmental risk [2]. In a large, population-based study, food-insecure children aged 2–5 years old had 1.57 times the odds of being diagnosed with a developmental delay and/or behavioral problems compared to those in food-secure households. Utilizing SNAP was a protective factor; there was no difference between food-insecure families that used SNAP and food-secure families [50]. Treviño et al. (2023) found that households that were food insecure or marginally food secure when their child was 3 years old had more developmental delays across many domains—communication, gross motor, fine motor, personal–social and problem solving—compared with households that only experienced food insecurity when their child was 1 years old or those that never experienced food insecurity [51]. Food insecurity is associated with a greater number of concerns on a parental developmental screening questionnaire spanning language, motor, behavior, social–emotional, self-help, and school readiness domains [52,53]. However, the research on food insecurity, specifically on gross and fine motor development delays, has mixed findings; a recent meta-analysis did not find an association between food insecurity and motor development [3].

### 3.3. Social–Emotional and Cognitive Functioning

One of the main functions of preschool is to prepare children for kindergarten and formal schooling, often called school readiness. Social–emotional and behavioral skills and competencies are a key component of school readiness [54]. Such social–emotional skills essential for learning in a kindergarten setting include playing well with others or being able to bounce back when things do not go their way. Examples of essential self-regulation skills include being able to stay focused and sit still during lessons. Food insecurity in the preschool years is associated with poorer school readiness, particularly in the self-regulation and social–emotional development domains [55]. In a study of preschool-aged children, Jackson et al. (2021) found that compared to food-secure children, those experiencing moderate-to-severe food insecurity had over twice the incidence rate of needing support for, or being at risk of, not developing social–emotional and self-regulatory skills [55].

Food insecurity in preschool is not only associated with concurrent school readiness skills but also predicts difficulty implementing these skills in kindergarten. A longitudinal study utilizing data from the Early Childhood Longitudinal Study Birth Cohort found that among a low-income sample, very low food security in preschool predicted more conduct problems (e.g., throwing tantrums) and poorer approaches to learning in kindergarten (e.g., eagerness to learn), even after controlling for food insecurity at nine months and two years of age. Persistent food insecurity throughout early childhood was correlated with hyperactivity in addition to conduct problems and poorer approaches to learning [56].

Social–emotional skills are also related to indicators of emotional health, such as externalizing (e.g., aggression and hyperactivity) and internalizing problems (e.g., anxiety and depression) [57]. Given research demonstrating that food insecurity disrupts the development of these skills [55,56], it is no surprise that food insecurity is associated with both internalizing and externalizing behaviors in preschoolers [58,59,60,61]. A recent meta-analysis on food insecurity and early childhood development found high heterogeneity among the studies investigating the association between food insecurity and emotional health indicators [3]. Despite the high amount of heterogeneity, meta-analyses revealed that food-insecure children are more likely to experience difficulties in these areas compared to their food-secure peers [3]. Treviño et al. (2023) found that children from food-insecure or marginally secure households experienced more problem behaviors than those from food-secure households [51].

Longitudinal studies indicate that food insecurity in early childhood can have lasting effects on these markers of emotional health. One study utilizing the Longitudinal Study of Child Development in Quebec found that compared to food-secure children, children who experienced food insecurity at one-and-a-half years and four-and-a-half years were more likely to have persistently high levels of depression/anxiety and hyperactivity/inattention through age eight [62]. However, only the association with hyperactivity/inattention persisted after controlling for poverty-related variables.

Evidence also suggests that food insecurity experienced in preschool can also impact cognitive development and academic achievement [55,56], although such effects may not be as strong as those for social–emotional outcomes [56]. Jackson et al. (2021) found that both mild and moderate-to-severe food security is associated with an increased rate of needing support or being at risk of not meeting early learning skill targets such as sounds, letters, and counting [55]. Johnson and Markowitz (2018) found that among a sample of low-income children, low food security experienced in preschool was associated with poorer reading skills in kindergarten [56]. Only one study has examined the relationship between food insecurity and executive function in preschoolers; Bryant et al. (2020) [63] found that poorer parent reports of the community food environment (access to, availability, and affordability of foods) were associated with poorer child performance on the Head-Toes-Knees-Shoulders task. However, when food access was measured via census data instead of parent reports, no significant association with executive function was found [63].

Why might food insecurity affect social–emotional and cognitive development, even if experienced transiently? While the findings between food insecurity and development reported here have statistically accounted for poverty, it remains that food insecurity is difficult to disentangle from other physical (e.g., substandard housing) and psychosocial stressors (e.g., household chaos), and these stressors may overwhelm children’s self-regulatory systems [64]. A large study of over 25,000 caregiver–child pairs found that as the number of stressors experienced increased, child health and development indicators worsened [53]. A review of the ways that poverty may impact neurodevelopment found considerable evidence that aspects of poverty and lower socioeconomic status (e.g., increased exposure to pollutants or waste and exposure to violence) are associated with increased behavior, attention, memory, and developmental delays [25]. It is possible, then, that the emotional health of children who are food insecure is more susceptible to disruption. For example, Lamptey et al. (2025) found that food-insecure families were more likely to report that their children were worried, lonely, and showed decreased happiness during the pandemic [65].

Social–emotional difficulties experienced in children may be, in part, due to stress and poor mental health among food-insecure parents [18,66]. The Family Stress Model [67] is one evidence-backed framework to conceptualize how stress on the family impacts child health and development [68]. This theory proposes that economic hardship, like food insecurity, leads to economic pressure, which leads to parental psychosocial stress [68]. This stress can result in less responsive caregiving [52], which is important for building secure parent–child attachments [69] and supporting child emotional and cognitive development [70]. For example, prospective studies have demonstrated that psychological distress (e.g., depression and anxiety) as influenced by economic stress is linked to reduced quantity and quality of time a parent spends engaging with children [71], lower levels of sensitivity and supportiveness [72], and fewer educational and enriching activities in the home environment [73].

Evidence suggests that the Family Stress Model can be applied to food-insecure parents and their children, including those who are marginally food secure [74]. These parents have higher stress than food-secure parents [18], are more likely to exhibit depressive symptoms [19], and their children are more likely to have behavior problems [60]. Psychosocial stress from food insecurity seems to at least partially explain some negative child development outcomes. For example, Nagata et al. (2019) found that the relationship between food insecurity at age four and oppositional defiant problems at age five was partially mediated by maternal depression [66]. Encinger et al. (2020) found that marginal food security had a negative, indirect effect on preschoolers’ self-regulatory skills via maternal depression [18]. However, it should be noted that some families are able to protect their children from the effects of parental stress with the help of certain coping strategies (e.g., emotion regulation and positive thinking) and social support [75].

Finally, nutrient inadequacies from food insecurity have across-the-board impacts on social–emotional and cognitive development. Iron is extremely important for neurodevelopment [76], and iron deficiency anemia is associated with deficits in social–emotional [77], cognitive [78], and immune function [79]. Additionally, with protein energy malnutrition, children may be more tired and less likely to engage with their physical and social environment, an important driver of social–emotional and cognitive development [80].

### 3.4. Limitations in Food Security and Child Development Research

The studies discussed in this section are cross-sectional or longitudinal in design. While longitudinal studies provide stronger evidence of a possible link between food security and child development, neither design provides clear evidence for causal pathways. While each study controlled for poverty via their statistical analyses or study design, studies varied in the number and type of additional confounding variables controlled for (e.g., race and maternal education). While it is a strength that food insecurity was most commonly measured with the HFSSM or adaptations of it, these findings therefore represent the association between child development outcomes and food insecurity as a function of limited financial resources. If food security were conceptualized more broadly, or other specific domains were explored, it may be possible to gain a more nuanced understanding of the ways food insecurity affects children. Additionally, while measures of child development outcomes are necessarily varied, this can make it difficult to synthesize the literature into concrete and conclusive findings. These shortcomings limit our ability to understand on a more granular level what specific aspects of child development are most likely to be impacted by food insecurity, and how.

## 4. The Evidence Behind Childcare as a Food Insecurity Intervention

Recent reviews have discussed the evidence supporting a range of interventions intended to reduce food insecurity in high-income countries, including federal and state-sponsored programs (e.g., SNAP and WIC) and community-level efforts (e.g., food banks) [5]. While these programs are important in the pursuit of radically reducing the prevalence of food insecurity in the US, the potential for childcare arrangements to be a point of intervention for food insecurity is not often discussed in the scientific literature. For example, the National School Lunch Program is often pointed to as the primary way children from food-insecure families are fed. However, what about young children who have not yet entered kindergarten? Research suggests that some childcare arrangements can reduce household food insecurity [7]. Childcare is well-positioned to address child food insecurity and positively impact child development in several ways: by supporting children’s cognitive and social–emotional development; providing breakfast, lunch, and snacks to the children they serve; responsively feeding children; and identifying and supporting families that are food insecure. This section reviews several ways that childcare can be leveraged to address food insecurity.

### 4.1. Supporting Child Development

Childcare may buffer the effect that food insecurity has on child development outcomes [56]. For example, Johnson and Markowitz found that kindergarten reading and math skills were more affected by food insecurity during infancy and toddlerhood than by food insecurity during the preschool years [56]. They theorized that this may be due to the extra food and cognitive stimulation that the child received in childcare [56,81]. However, not all childcare arrangements are equally as effective at mitigating risk and promoting development; evidence suggests that classroom quality as well as teacher and child characteristics determine the direction and strength of the relationship between childcare and positive outcomes [81,82,83,84,85].

### 4.2. Providing Food via the Child and Adult Care Food Program

While all children eat at childcare, how the food is provided varies in the United States. Many childcare providers do not provide food, and parents are instead required to pack food for their child [86]. Not only may food-insecure parents have difficulty packing enough food for their children, but it also may not be of high nutritional quality. Packed lunches often do not provide at least a third of the daily recommendation for energy, carbohydrates, a range of essential micronutrients, fiber, fruits and vegetables, or milk [87]. Additionally, parent-targeted interventions to improve such lunches are only marginally successful; parents persist in packing small amounts of vegetables and large amounts of sweets and chips [88]. Further, when children eat parent-provided food, teachers tend to use more controlling feeding practices than when the center provides the food [89].

The Child and Adult Care Food Program (CACFP) is a federal program that provides reimbursements for meals and snacks aligning with national nutrition guidance that are served to eligible children and adults in their care at participating childcare centers, day care homes, adult day care centers, afterschool care programs, and emergency shelters [90]. Like other federal food assistance programs, CACFP eligibility criteria are largely based on household income and/or enrollment in other federal assistance programs. Therefore, the level of reimbursement that a childcare provider can receive is determined by the number of children whose families are below the maximum income limit, which requires collecting income information from families Head Start programs receive full reimbursement for all children, as they are categorically eligible [90]. Compared with childcare that does not participate in CACFP, those that do participate (including Head Start centers) serve more fresh fruit, whole grains, and low-fat milk. CACFP centers also use more responsive feeding practices, such as family-style meals and modeling eating healthy food, and avoiding the use of controlling feeding practices [91,92,93]. CACFP has been shown to reduce household food insecurity [7,94,95] and the risk of being underweight or overweight, as well as improve dietary quality [91,94,96]. Children attending CACFP-participating childcare ate more vegetables, fruit, whole grains, and dairy, and less saturated fats and added sugars on days they attended childcare than days they did not attend [97].

However, CACFP is not effectively reaching low-income and likely food-insecure children. Among preschool-aged, low-income children, only 37% receive CACFP-reimbursed meals and snacks in childcare [98]. Additionally, children from low-income households who live in high-income areas are especially unlikely to receive CACFP-reimbursed meals [98]. One reason for this is that very few states allow license-exempt home-based childcare providers to participate in CACFP, even though it is allowed under federal law. Small home-based providers or friends and relatives that are legally exempt from state childcare licensing requirements constitute the most common form of childcare in the US, and are disproportionately utilized by the most vulnerable families [98,99], such as children from immigrant families, children with disabilities, families who cannot afford childcare, or families working non-traditional hours [100]. However, even states that do allow these forms of childcare to utilize CACFP may not actively recruit these providers because of convoluted approval, enrollment, and training processes that involve both the provider and the parent they are serving, and because funds for the administrative support of these providers are limited [101].

In addition to restrictive eligibility criteria, a low awareness of the program, low reimbursement rates, significant barriers to entry, and high administrative burden may be driving low CACFP enrollment. In a survey of 256 non-CACFP centers that served meals or snacks, 52% had never heard of CACFP [91]. To receive CACFP benefits, sites are required to be supported by a “sponsor”, a registered non-profit organization that helps sites receive and utilize their benefits according to the regulations. While childcare centers that have more robust administrative capabilities can be their own sponsor, smaller childcare providers (e.g., family home-care providers) must find an external sponsor. This may be a significant barrier to enrollment; providers are more likely to enroll in CACFP if they are a part of a larger network of childcare providers or receive Head Start referrals [98].

Participating in CACFP comes at a high administrative cost. Andreyeva et al. (2024) found that 80% of the state agencies interviewed mentioned paperwork burden as a barrier to CACFP participation [102]. Navigating the eligibility criteria is difficult for providers, especially family home-care providers who do not have a large administrative support team and those located in non-urban areas [96,103]. And, if meals are not documented appropriately, providers may not receive CACFP reimbursement. Further, meal reimbursement amounts from CACFP do not cover food expenses entirely for eligible children [97]. Child hunger advocates have suggested that it is for these reasons that providers may forgo CACFP reimbursement entirely, and instead rely on parent-provided food, or cheaper, less nutritious options [104].

### 4.3. Providing Food via Other Methods

Because of the difficulties in implementing CACFP, childcare providers may provide food to children and their families in other ways. Creative local-level partnerships have been employed to leverage the frequent touchpoints and relationships that childcare providers have with parents. For example, in North Carolina, a local health authority partnered with childcare providers to offer pre-prepared, healthy, and low-cost meals to families [105]. Some centers are partnering with food banks to provide extra food or to provide backpacks of food for weekends or long breaks [106]. Food insecurity and transportation insecurity may coincide [107]; one childcare center found that they were best able to serve families when the food pantry was co-located with the childcare center and could be easily accessed by administrators and teachers [108].

There is limited research on how co-located food pantries, or “backpack” programs in which food is sent home with parents for the weekend or holidays, may impact food insecurity specifically in childcare settings. However, research suggests that elementary school backpack programs may reduce household food insecurity and increase attendance, especially on Fridays when backpacks are distributed [109,110]. In a pilot of a combined school-based food pantry and backpack program, parents reported that they were happy with both approaches but could feed more family members with the pantries than the backpack program. These approaches may help bridge the gap in food accessibility during tough times of the month. One participant said, “It actually gets a lot of families through on their tougher times… Some of us won’t get our food stamps until later. I don’t get my food stamps until the 14th and his dad isn’t always able to get enough groceries in the house. So, when they send them home, it actually helps out” [111]. It is important to note, however, that some have voiced criticism of backpack programs; they may have unintended consequences such as shame, stigma, and disruptions to family functioning, and they obviate the need for structural, long-term strategies [112].

### 4.4. Training Childcare Providers to Support Food-Insecure Children in the Classroom

Teachers may be concerned about their students not receiving enough food at home, but they may struggle with how to respond [106,113,114]. Care providers notice children’s voracious appetites, and that some are arriving hungry at childcare, especially after weekends or long breaks [106,113,114]. One teacher from Swindle et al.’s [114] study says:

“And we have some that come in and say, I didn’t get to eat last night. Or they’ll want more, seconds. If we have seconds, you know, if we have seconds we give it to them and, um, we have one little girl in particular that she’ll come in sometimes on Mondays and she’ll eat three or four helpings of something. And we’re pretty sure, you know, she might not have ate a lot over the weekend.”

Teachers report that center policies do not meet the needs of children, so they develop their own, personal strategies to manage their concerns [114]. Many of these strategies revolve around trying to obtain extra food for the children at the center. However, providing enough food for these children can be challenging because CACFP allows only one entrée per child, regardless of food security status. In efforts to keep costs down, some centers may prepare more lower-cost foods like rice or potatoes to supplement when a child asks for more food [106]. Teachers may give the child food off their own plate or personally go to the kitchen to try and find additional food [114]. In a survey of over 1500 Head Start programs, 37% reported keeping additional food on hand to feed children perceived to be food insecure [115].

Additionally, childcare providers, like parents, may use more controlling feeding practices, particularly if they themselves are food insecure [114,116]. Early childhood education teachers and care providers are important role models for healthy eating behaviors, but modeling can be difficult in the context of food insecurity. Compared to food-secure educators, educators with current or previous experience with food insecurity are more concerned that their students are not eating enough and use more controlling strategies, such as pressuring them to eat [114,117]. For example, one teacher said of feeding her students:

“We try to really, really get them to eat because sometimes these are the only meals the kids eat. And if we have gave them the amount we’re supposed to, serving size … We know they’re hungry, we’re going to give them more, you know” [117].

Teachers may struggle to prioritize exposure to new and healthy foods (e.g., vegetables) over familiar foods because of their desire to ensure that children eat that day. One teacher from Swindle et al. [114] said:

“And it still brings up that inner struggle ‘fill their belly or expose them to new foods?’ That’s like the biggest thing that stuck out with me now, because you want to fill their belly. And is it more important to feed them something healthy or make sure they go home with something in their stomach?”

Some childcare providers must adhere to certain health program standards. For example, Head Start programs have performance standards that require certain feeding practices, such as teachers sitting with children during mealtime, serving meals family-style (where food is presented in the center of the table, and children can serve themselves the amount they want), and refraining from using controlling feeding practices [118]. It is likely due to these performance standards that Head Start programs more frequently employ these practices [119]. Early childhood programs accredited by the National Association for the Education of Young Children (NAEYC) must meet a health program standard that includes promoting the nutrition and health of children and staff [120]. Not everyone meets this standard and receives accreditation; a recent analysis found that the scores on the health program standard were the most predictive of accreditation and that there was more variation in these scores than the other program standards [121].

### 4.5. Screening and Supporting Food-Insecure Families

Childcare centers may also help connect families experiencing food insecurity to resources. Head Start centers often connect families with food assistance programs and resources [115]. Similarly, CACFP centers are more likely to screen for families’ food security status than non-CACFP centers [122]. However, not all childcare providers may feel comfortable doing this. In a study of 1600 centers across four states, less than half of the teachers who reported having hungry children in their classroom broached the question with parents, but the vast majority of teachers brought the issue to their center director [106]. Center directors may also be reluctant to bring this issue up to the parents because they consider it to be a private family issue [122]. Similarly, interviews with childcare providers demonstrate that they may be reluctant to discuss students’ nutrition with parents because parents may be too busy to talk, providers are unsure how to discuss nutrition without offending parents, or do not know if parents are receptive to nutrition education resources [123].

While there are limited data on the effectiveness of food security screening in childcare contexts, screening has been effective in both identifying and referring patients to resources in medical settings [124]. One randomized controlled trial testing an intervention designed to screen families for a range of psychosocial issues, including food insecurity at primary care visits, found that patients in the intervention group reported discussing food insecurity in 25% of the visits, compared to only 8.4% in the control group. Additionally, 12% received a referral to food resources like federal assistance programs and food pantries, while only one percent received a referral in the control group [125]. A pilot program that embedded a food-insecurity resource navigation program into pediatric primary care, with the goal of connecting patients to community-based food assistance resources, saw incremental shifts towards food security 3 and 6 months after the initial service [126].

## 5. Conclusions and Recommendations for Research, Policy, and Practice

There are far more children experiencing varying levels of food insecurity than is currently reported in the US [11], and its effects on child health and development across physical, social–emotional, and cognitive domains are concerning [2,3]. While evidence supports childcare as an important lever to pull when addressing food insecurity in the US, we recognize that better childcare is not the only solution. Other literature reviews have reported the evidence behind federal and state programs like SNAP, WIC, and the National School Lunch Program [5]. These programs are effective and critical to addressing food insecurity experienced by all children in the US, and research demonstrates that food banks alone are not enough to meet nutrition or individual needs [127]. Yet, political sentiment towards food-assistance is increasingly negative. Already, legislation passed by the 118th U.S. Congress markedly reduces funding for SNAP and limits enrollment. Because the cost of SNAP will be passed to the states, states are already cutting other food assistance programs to continue operating SNAP. Funding for high-quality childcare, CACFP, and other programs that serve young children should not be taken for granted, and may play a more important role than ever as other programs are less able to serve food-insecure families. Without significant advocacy, food insecurity may continue to worsen in the coming years as changes take effect.

The purpose of this section is to make evidence-based recommendations for how researchers, policymakers, and childcare providers can support (1) a further understanding of how food insecurity affects child development, (2) further understanding of how childcare may address food insecurity, and (3) strengthen childcare as an intervention for food-insecure families with young children.

### 5.1. Further Understanding of the Effect of Food Insecurity on Child Development

Researchers should continue to investigate the effects of marginal food security on families and child development in addition to other more severe forms of food insecurity (Recommendation 1, Table 1). Food security measurement should capture the psychosocial aspects of food insecurity, either by evolving the HFSSM or using other measures alongside it, to help craft thoughtful interventions that adequately address families’ needs (Recommendation 2, Table 1).

It is evident that early experiences of food insecurity impact child development across physical, cognitive, and social–emotional domains [2,3]. However, additional research, particularly prospective longitudinal studies, will be important for understanding the more nuanced ways that food insecurity impacts child development. In particular, longitudinal research that can measure the temporal dynamics of food insecurity and how it impacts dietary quality, feeding practices, and cardiometabolic outcomes is needed (Recommendation 3, Table 1). Additionally, to further understand the differential impact that food insecurity has on social–emotional versus cognitive domains, future research should take a more nuanced approach and investigate how food insecurity impacts certain social–emotional skills (e.g., emotion regulation) and cognitive skills (e.g., executive function), rather than focusing solely on behavior problems (e.g., internalizing/externalizing) and academic outcomes (Recommendation 4, Table 1).

### 5.2. Further Understanding of How Childcare and Related Food Assistance May Address Food Insecurity

Changing administrative priorities pose threats to Head Start and other childcare funding at the federal level [128,129]. It is important that affordable, high-quality childcare is available to food-insecure families and those living in poverty. Advocacy at the state and federal level is urgently needed to preserve and expand funding for high-quality childcare (Recommendation 5, Table 1). ChildCare Aware of America and NAEYC are two such organizations leading advocacy in this area. More research on how quality childcare may be able to buffer the effects of food insecurity and policy is imperative (Recommendation 6, Table 1).

It is also important to continue to evaluate and share the results of food-security-focused interventions in childcare for their cultural appropriateness, effectiveness, and sustainability (Recommendation 7, Table 1). Results of pilot programs and small projects are helpful in lending support to potentially larger projects and policy solutions. Researchers investigating food assistance programs like CACFP should share their findings with advocacy organizations and with policymakers directly. Data on how CACFP and childcare may reduce food insecurity, support child development, and yield long-term return on investments are important to convince policymakers to devote more resources to research and program implementation. Similarly, childcare providers can share their stories serving food-insecure families or invite policymakers to visit their childcare to witness the nutritious and cost-effective meals and the high-quality care they provide.

### 5.3. Strengthen Childcare as an Intervention for Food-Insecure Families with Young Children

With the help of CACFP, childcare providers can serve nutritious food to food-insecure children without passing the cost along to parents. Funding for food assistance programs, including CACFP, should not be taken for granted and will need to be supported in the evolving political climate. Continued funding as well as several changes to CACFP are needed to promote enrollment, maintain participation, and provide enough food to food-insecure children, including revising eligibility criteria, reducing paperwork requirements, increasing reimbursement rates, and reimbursing childcare providers an additional meal (Recommendation 8, Table 1). Advocacy leaders such as the Food Research Action Center, CACFP Matters, and the National CACFP Sponsors Association are leading advocacy, especially at the federal level. State agencies can improve access to CACFP by extending enrollment to license-exempt home-based childcare providers, simplifying approval processes, and not requiring anything beyond federal requirements [102]. State action may be particularly important in times when the federal government does not prioritize the improvement of safety-net programs.

Until systemic changes to CACFP can be made, childcare providers may benefit from utilizing the training and resources offered by the government and private sector organizations (Recommendation 9, Table 1) [130]. Given the reductions in the federal workforce, private sector organizations may be increasingly important for recruiting and providing support to childcare providers as they implement CACFP. For example, the National CACFP Association conducts recruitment activities, connects providers to sponsors, offers resources (e.g., meal planning), certification programs, and training (e.g., reimbursement rates for certain foods) to those who administer, operate, and participate in CACFP [131].

Especially when CACFP participation is not possible, particularly in the case of unlicensed care providers in many states, childcare providers should consider creative ways to provide families with additional food (Recommendation 10, Table 1), such as arrangements with local food pantries, sponsoring organizations, or backpack programs. As childcare centers identify ways to serve their families, it will be important to collaborate with parents to determine if these methods are indeed the best way to assist food-insecure families in the long run and to ensure that it is executed in a way that mitigates any unintended consequences.

Childcare providers should develop center-wide or personal feeding policies and strategies for feeding children who appear to be very hungry (Recommendation 11, Table 1). Head Start programs already operate under performance standards that require certain feeding practices [118], and NAEYC accreditation program standards require programs to document nutrition practices and share them with staff and families [120]. Such policies do not specifically include guidance about feeding food-insecure children, but can serve as a starting point for all types of childcare providers who aim to implement more specific feeding policies. Providing teachers with additional strategies for feeding hungry children may help take the guesswork out of how to attend to these children’s needs and prevent the use of controlling feeding practices. Such strategies could include guidance about how much, what kinds, and when teachers can provide extra food, refraining from pressuring a suspected food-insecure child to eat during mealtimes.

Childcare arrangements can leverage the many touchpoints they have with families to implement food-security screening and provide resources for accessing federal assistance programs or food banks to all families, regardless of food security status (Recommendation 12, Table 1). Head Start programs and many CACFP-funded programs screen families for food insecurity [115,122], but many other programs do not, given the sensitive nature of food security status. While food insecurity is not specifically mentioned, NAEYC accreditation standards emphasize cultivating family relationships and providing information about other organizations and services [120]. Providers should leverage this relationship to share what food services are available in the community. Engaging with families about food and nutrition can be difficult, but research suggests that when providers foster respectful relationships with families centered on supporting their child, frequently connect with parents during and outside of pick-up/drop-off times, and discuss center policies (e.g., universal food security screening, state and federal nutritional standards, responsive feeding), providers may be able to discuss the child’s food and nutrition in the home environment and share the appropriate resources [123].

Food insecurity has a profound impact on child outcomes, even in high-income countries like the United States. Food insecurity works together with, and separately from, poverty to threaten child health and development across physical, social–emotional, and cognitive domains. Childcare providers have the unique opportunity to provide both enrichment and nourishment to children and to leverage their daily connection with parents to provide them with and connect them to resources. Strengthening childcare is an overlooked opportunity to address the increasing rates of food insecurity among households with children in the US, and action from all stakeholders—government, policymakers, researchers, and childcare providers—is necessary to ensure childcare providers can serve food-insecure families to the greatest extent possible.

## Figures and Tables

**Table 1:** Summary of recommendations.

	Recommendation	Stakeholder
1	Investigate the effects of marginal food security in addition to more severe forms	Researchers
2	Measure psychosocial aspects of food insecurity to inform interventions	Researchers
3	Conduct longitudinal research that measures temporal dynamics of food insecurity	Researchers
4	Conduct research that measures impact of food insecurity on specific social–emotional and cognitive skills	Researchers
5	Ensure access to affordable, high-quality childcare	Researchers
6	Conduct research that investigates the role of quality childcare in buffering the effects of food insecurity	Policymakers
7	Evaluate and/or share the results of food-security-focused interventions in childcare for their cultural appropriateness, effectiveness, and sustainability	Policymakers, Researchers, Childcare providers
8	Continue to fund CACFP, and change key elements of CACFP (eligibility criteria, paperwork burden, and number of reimbursable meals)	Policymakers
9	Utilize training offerings from government and private-sector sources to support CACFP implementation	Childcare providers
10	Consider creative ways to provide families with additional food	Childcare providers
11	Provide specific feeding guidance and strategies to teachers for feeding children who appear to be very hungry	Childcare providers
12	Leverage and create touchpoints with families to implement food security screening and provide resources	Childcare providers

## Data Availability

No new data were created or analyzed in this study. Data sharing is not applicable to this article.

## Abbreviations

The following abbreviations are used in this manuscript:

SNAP	Supplemental Nutrition Assistance Program
CACFP	Child and Adult Care Food Program
HFSSM	Household Food Security Survey Module
NAEYC	National Association for the Education of Young Children
